# Cytokine profiling in anti neutrophil cytoplasmic antibody-associated vasculitis: a cross-sectional cohort study

**DOI:** 10.1007/s00296-019-04364-y

**Published:** 2019-07-08

**Authors:** Johanna Charlotte Hoffmann, Daniel Patschan, Hassan Dihazi, Claudia Müller, Katrin Schwarze, Elvira Henze, Oliver Ritter, Gerhard Anton Müller, Susann Patschan

**Affiliations:** 1 University Hospital Göttingen, Clinic of Nephrology and Rheumatology, Göttingen, Germany; 2 Zentrum für Innere Medizin 1, Cardiology, Angiology, Nephrology, Klinikum Brandenburg, Medizinische Hochschule Brandenburg, Hochstraße 29, 14770 Brandenburg, Germany

**Keywords:** AAV, Cytokines, VDI, BVAS

## Abstract

ANCA-associated vasculitides (AAV) are severe diseases, potentially affecting lungs, kidney, and other organs. Nevertheless, risk profiling remains difficult. Aim of the current study was to analyze serological characteristics in AAV. The principal goal was to identify diagnostic markers that potentially allow a more sophisticated risk profiling in AAV. AAV subjects were recruited and evaluated for disease activity, disease stage, medication, and laboratory findings. Serum concentrations of the following parameters were measured: IL-1β, IL-6, IL-17 A, IL-17 F, IL-21, IL-22, IL-23, TNF-α, sCD40L, IL-4, IL-10, IL-25, IL-31, IL-33, and INF-γ. A total number of 62 AAV subjects was included in the study (39 females; 23 males). Forty-five subjects were PR3+, 17 subjects showed ANCA specificity for MPO. The majority of all cytokines fell under the lower detection limit of the assay. Serum IL-10 was higher in both, AAV and SSc as compared to controls; it was also higher in early systemic AAV. Serum IL-33 was elevated in AAV and SSc; in AAV, higher levels were found in non-necrotizing GN and RTX untreated subjects. Serum CD40L was raised in AAV as well; higher concentrations were also found in PR3+ and MPO+ patients and early systemic, generalized, and refractory AAV. IL-10 may potentially serve as a marker of early systemic AAV. IL-33 may help to identify subjects with a higher risk for necrotizing GN in AAV.

## Introduction

ANCA-associated vasculitides (AAV) are the most frequent types of primary small-vessel vasculitides, according to the revised Chapel Hill consensus conference nomenclature from 2012 [1]. At least three distinct disorders represent AAV, each characterized by inflammatory damage of small blood vessels including arterioles, capillaries and venules, and each associated with peripheral circulating anti-neutrophil cytoplasmic antibodies (ANCA) of different antigen affinity. Granulomatosis with polyangiitis (GPA) typically affects lung and kidney, accompanied by the formation of granulomas in a locally destroying manner. Numerous other organs may be damaged, as well [2]. ANCA in GPA predominantly interacts with cytoplasmic proteinase 3 in granulocytes (PR3-ANCA). Antibodies may be absent during earlier disease stages but can be detected in more than 90% if the disease generalizes [3]. Clinically, microscopic polyangitis (MPA) is almost indistinguishable from GPA. However, granulomas are absent, and ANCA mostly interacts with the perinuclear antigen myeloperoxidase (MPO-ANCA) [2]. The third and least frequent type of AAV is eosinophilic granulomatosis with polyangiitis (EGPA) [4]. EGPA patients suffer from allergic rhinitis/asthma and show enrichment of eosinophils in tissues and blood, findings that have been summarized in the current classification criteria [5]. Finally, renal-limited ANCA-associated vasculitis may be considered the fourth entity of AAV, as suggested by Pagnoux [2]. The prognosis of AAV patients often depends on rapidly initiated diagnostic steps helpful to identify possible end-organ damage. Before the era of intensified immunosuppressive treatment using steroids and cyclophosphamide combined [6], more than 90% of all patients presenting with kidney failure and lung involvement (pulmonary-renal syndrome) died from the disease(s). This situation has significantly been improved in recent years, last but not least, with the introduction of rituximab as a therapeutic measure in generalized and remitting GPA/MPA [7]. Nevertheless, even if the diagnosis is correct and immunomodulatory therapy has been initiated, the individual treatment response remains challenging to predict. For instance, refractory retroorbital manifestations have been shown to respond less sensitive to drug therapy than resistant glomerulonephritis [8].

Current diagnostics for identifying the disease per se and for monitoring patients during treatment include history, clinical examination, radiographic analyses, urine analyses, and ANCA testing. More disease-specific laboratory parameters are still missing. Especially markers with substantial predictive potency in terms of disease severity/activity and the risk for chronic damage are urgently needed. Also, the individual sensitivity towards certain types of immunosuppressive drugs is hardly predictable. It also remains unclear how subjects with higher relapse risk may be identified in advance and how such information can be transferred into the clinical management of AAV.

Therefore, the current study aimed to screen serological characteristics in AAV patients. We intended to evaluate individual serum cytokines in subjects with different epidemiological and clinical characteristics, particularly with varying severity of end-organ involvement. The principal goal was to detect future diagnostic candidates that allow a more sophisticated/reliable risk profiling in AAV.

## Methods

### The setting, study population and study criteria

All participants were recruited from the Clinic of Nephrology and Rheumatology of the University Hospital Göttingen (Germany). The local ethics committee approved the study (name: ´ethics committee of the Universitätsmedizin Göttingen; Approval Number: 09/10/15; date of approval: October 2015). Inclusion criteria: subjects with newly diagnosed or established AAV (GPA was initially classified according to the 1990 published criteria of the American College of Rheumatology [9]. Later, we employed revised criteria which have been introduced at the annual meeting of the American College of Rheumatology, held in Washington DC on 11/14/2016 [Raashid Luqmani (University of Oxford), Peter A. Merkel (University of Pennsylvania), Richard Watts (University of East Anglia)]. The new classification incorporates nine individual criteria, encompassing clinical and laboratory findings with either positive or negative predictive power. A total score of 5 or higher indicates the disease with high probability), age > 18 and < 90 years; exclusion criteria: malignant disorder, uncontrolled infection at the time of inclusion. The participants signed consent for the data to be analyzed and published. The fact that every patient fulfilling the respective criteria was included if she/he signed the consent form potentially reduced selection bias.

Numerous clinical and laboratory parameters were collected from each subject including history and physical examination, cardiovascular risk profile (e.g., family history, arterial hypertension, diabetes, smoking habits), average alcohol consumption, and nutritional state. Also, ANCA-associated organ involvement was documented, particularly involvement of upper/lower respiratory tract, kidney, skin, joints, and nervous system. Renal involvement was defined as biopsy-proven glomerulonephritis, a biopsy was either performed due to de-novo acute kidney injury as described in the latest version of the KDIGO criteria [10] or due to significant glomerular proteinuria. The disease activity was quantified using the Birmingham Vasculitis Activity Score (BVAS) [11] with a score of < 8 indicating low activity as opposed to ≥8 as indicative for high activity. Irreversible organ damage was evaluated using the Vasculitis Damage Index (VDI) [12]. AAV staging was performed according to 2007 published EULAR recommendations [13]: localized, early systemic, generalized, life-threatening, and refractory.

The control group included age- and gender-matched individuals with no known autoimmune-mediated disorder. Subjects were recruited from the staff of the University Hospital of Götingen. Thus, it was ensured that they matched the cohort of interest (AAV). The second control group included patients with systemic sclerosis (SSc). They were also recruited from the Clinic of Nephrology and Rheumatology of the University Hospital Göttingen (Germany).

### Serological analysis

Serum concentrations of the following parameters were measured using the Bio-Plex Multiplex Immunoassay System (BioRad) according to the manufacturer’s instructions: pro-inflammatory cytokines—IL-1β, IL-6, IL-17 A, IL-17 F, IL-21, IL-22, IL-23, TNF-α, and sCD40L; anti-inflammatory cytokines—IL-4, IL-10, IL-25, IL-31, IL-33, and INF-γ. The cytokine analysis was performed once, at the time of study inclusion.

### Statistical analyses

All analyses were performed using the application STATISTICA (StatSoft). Data subsets underwent distribution analysis using the Kolmogorov–Smirnov test. Normal distribution was assumed if the respective *p* value was ≥0.05. Differences between two non-nominal data groups were calculated with the Mann–Whitney test for not normally distributed data and with the student’s t test for normally distributed data. Differences between nominal data were calculated with the Chi-squared test. Correlation analysis was performed by calculating the Pearson correlation coefficient. Differences between more than two groups were calculated using the ANOVA test. Differences were considered significant if *p* values were below 0.05.

## Results

In the first section, we will shortly summarize the clinical characteristics of the subjects; the second part will address serological abnormalities, particularly serum levels of specific pro- and anti-inflammatory cytokines. We will exclusively name differences that fulfilled the criteria of statistical significance.

### Clinical characteristics

#### Patients

Over a period of 1.5 years, we included 62 individuals with newly diagnosed or established AAV (newly diagnosed 16, established AAV 46; 39 females, 23 males), the age ranging from 24 to 83 years. Forty-five subjects were PR3+, 17 subjects showed ANCA specifity for MPO.

The mean age of all subjects was 60.5 years (females 60.9 and males 59.8 years), with a range of 24–86 years. The mean overall duration of the disease was 4.5 years; the range was 2 months to 22 years. The patients’ clinical characteristics are summarized in Table 1.

**Table 1:** Baseline epidemiological and clinical data of all included AAV individuals

No.	Age	Gender	Hypertension (y/n)	Diabetes (y/n)	eGFR < vs. > 60 ml/min	CRP (mg/dl)
1	70	M	y	n	>	7.5
2	82	M	y	n	<	124.9
3	54	F	y	y	>	109.8
4	55	F	n	n	>	245.6
5	58	M	y	n	<	150
6	86	F	y	y	<	94.3
7	74	F	y	n	>	2.9
8	72	F	y	y	<	96.4
9	70	F	y	n	<	12.3
10	79	F	y	n	<	129
11	48	F	y	n	<	2.9
12	45	F	y	n	>	77.2
13	72	F	y	n	>	150
14	61	M	n	n	>	81.3
15	64	F	y	n	<	45.7
16	45	M	y	n	>	218
17	71	F	y	n	>	31
18	43	M	n	n	>	0.8
19	67	M	n	n	>	42
20	56	M	y	n	>	49.3
21	59	M	n	n	<	96.1
22	74	M	y	n	>	5.4
23	66	F	n	n	>	2.5
24	49	F	n	n	>	0.7
25	45	M	n	n	>	2.7
26	79	F	y	n	>	4.3
27	73	M	y	n	>	2.2
28	65	M	y	n	>	<3
29	44	F	n	n	>	7.9
30	48	M	y	n	>	84
31	54	F	y	n	>	8.8
32	66	F	y	n	<	125.7
33	66	F	n	n	>	117
34	82	M	y	n	<	185.8
35	67	F	y	n	<	74.2
36	76	M	y	y	>	144
37	71	F	y	n	<	18.8
38	60	F	y	n	>	248
39	63	F	n	n	>	184.4
40	37	F	y	y	<	0.9
41	29	F	n	n	>	1.1
42	76	M	n	n	>	<2.0
43	61	F	y	n	<	1.8
44	48	F	n	n	>	0.6
45	41	F	n	n	>	1.5
46	53	F	n	n	>	47.2
47	73	F	y	n	<	31
48	71	F	y	y	>	134.1
49	59	M	y	n	>	197
50	76	F	n	n	>	N/A
51	58	F	y	n	>	89.8
52	27	M	n	n	>	1.7
53	68	M	y	y	<	45.6
54	45	F	y	y	<	162.9
55	32	M	y	n	>	5.8
56	42	M	n	n	>	166.3
57	66	F	n	y	>	33.1
58	49	F	n	n	>	22.2
59	55	M	y	n	<	101
60	24	F	n	n	>	13.9
61	75	F	n	y	>	9.2
62	79	F	y	n	>	111.1

*F* female, *M* male, *y* yes, *n* no, *N/A* not available

#### Disease activity

**Fig. 1:**
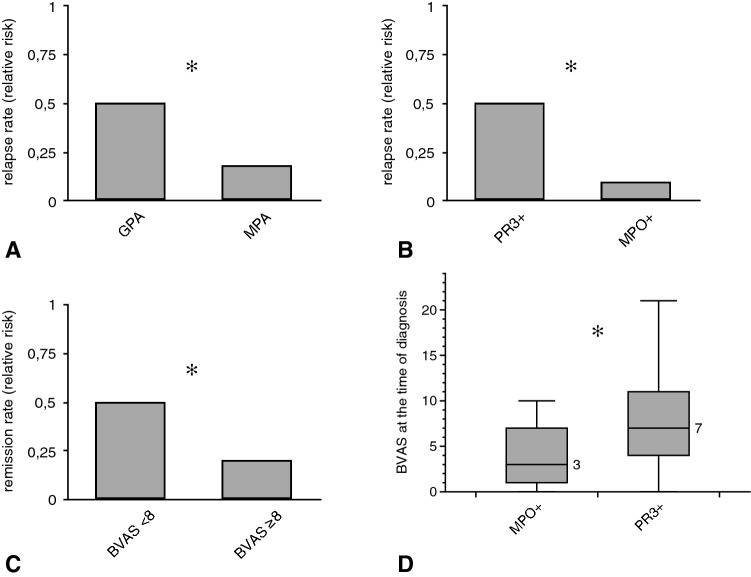
Disease activity-related parameters in GPA and MPA. The figure exclusively includes analyses with significant differences between the respective categories. **a** Relapse probability in GPA as compared to MPA, the results are depicted as relative risk with 1 reflecting a 100% relapse probability. The relapse probability was higher in GPA than in MPA. **b** since all GPA individuals were PR3+ and only one subjects with MPO positivity was diagnosed with GPA, the relapse probability was significantly higher in PR3+ as compared to MPO+ patients. **c** remission probability in relation to the mean BVAS. A higher likelihood for disease resolution was found in individuals with a BVAS of below 8 as compared to those with above 8. **d** patients with PR3 positivity displayed a higher mean BVAS at the time of diagnosis than MPO+ subjects (Kolmogorov–Smirnov test for normality: BVAS—*p* < 0.001; Data in **d** as media ± Q1/Q3; ✻*p* < 0.05—for exact *p* values see text)

In general, the relapse rate was higher in GPA than in MPA (50 vs. 18%; *p* = 0.02) (Fig. 1). At the time of diagnosis, PR3-ANCA + patients displayed a higher BVAS than MPO-ANCA + subjects (14.1 ± 6.9 vs. 8.7 ± 6.7; *p* < 0.001) (Fig. 1).Overall, PR3-ANCA + patients showed a higher relapse rate as compared to MPO-ANCA + subjects (50 vs. 10%; *p* = 0.01) (Fig. 1). A BVAS of higher vs. lower 8 and a VDI of higher vs. lower 1 were both associated with a higher relapse rate (both *p* values 0.001). However, a BVAS of above eight was associated with a higher remission probability (complete and incomplete − 50 vs. 20%; *p* = 0.01) (Fig. 1).

#### Renal involvement and kidney morphology

**Fig. 2:**
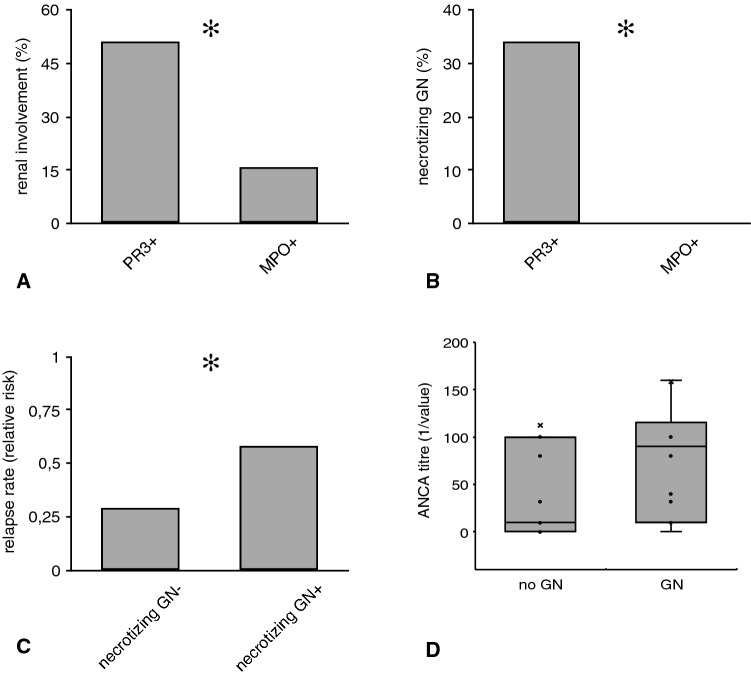
Renal involvement in AAV subjects. **a** PR3+ and MPO+ individuals in comparison. **b** Percentage of biopsy-proven necrotizing in PR3+ as opposed to MPO+ subjects. **c** Individuals with biopsy-proven necrotizing GN showed a higher relapse probability than those without the respective diagnosis. **d** Finally, subjects with necrotizing GN displayed higher average ANCA titers (Kolmogorov–Smirnov test for normality: ANCA titer—*p* < 0.001; Data in **d** as media ± Q1/Q3; ✻*p* < 0.05—for exact *p* values see text)

Kidney biopsy was successfully performed in 25 individuals. Overall, renal involvement occurred significantly more frequent in GPA than in MPA [23 (51%) vs. 2 (16%), *p* = 0.007] (Fig. 2). Also, kidney biopsy revealed necrotizing glomerulonephritis more frequently in GPA than in MPA [8 (34%) vs. 0 (0%), *p* = 0.004] (Fig. 2). The relapse rate was higher in individuals with biopsy-proven glomerulonephritis (58 vs. 29%, *p* = 0.02) (Fig. 2).

#### Respiratory involvement

Involvement of the upper respiratory tract was diagnosed in 37 patients (59%), manifestations were either rhinitis or sinusitis or otitis media. Six patients (9%) revealed sinusoidal granuloma formation. Individuals without pulmonary granuloma showed a lower relapse rate than those with granuloma formation (32 vs. 73%; *p* = 0.004).

#### Comorbidities

Patients with diabetes mellitus (*n* = 11), arterial hypertension (*n* = 39), hypercholesterolemia (*n* = 15) or asthma (*n* = 5) did not show higher relapse rates nor did they differ in terms of remission incidences from individuals without such disorders. Patients with Hashimoto’s disease (*n* = 11), however, showed a higher remission rate as opposed to subjects without the thyroidal disease (0.81 ± 0.4 vs. 0.37 ± 0.48; *p* = 0.006).

#### Drug therapy

The histological finding of necrotizing glomerulonephritis was associated with a higher cumulative dose of cyclophosphamide (8.610 ± 2.627 vs. 6.537 ± 2.213 mg; *p* = 0.02). Patients with an initial VDI of above 1 required immunosuppressive therapy for relapse control significantly more frequent than those below 1 (*p* = 0.01).

Patients receiving thyroid hormone due to Hashimoto’s disease benefitted more frequently from partial or complete remission (84 vs. 35%; *p* = 0.001).

### Serological characteristics

As described in the methods section, serological analyses were performed in AAV and two further cohorts, namely in patients with systemic sclerosis (*n* = 6) and in healthy subjects (*n* = 20), respectively. The following pro-inflammatory cytokines were quantified: IL-1β, IL-6, IL-17 A, IL-17 F, IL-21, IL-22, IL-23, TNF-α, and sCD40L. Anti-inflammatory cytokines included in the analyses were IL-4, IL-10, IL-25, IL-31, IL-33, and INF-γ. However, numerous cytokines could not finally be incorporated in the study since at least 50% of all quantified serum concentrations fell below the detection limit of the assay. Nevertheless, specific cytokines which were detected in such low levels in AAV were significantly elevated in systemic sclerosis: IL-4, IL-6, IL-17A, and IL-22. Thus, the following mediators will not be discussed in AAV: IL-1β, IL-4, IL-6, IL-17A, IL-17F, IL-21, IL-22, IL-23, IL-25, IL-31, INF-γ. Regarding AAV we will mainly address IL-10 (62% of all measured values within range), IL-33 (79% of all measured values within range), sCD40L (100% of all measured values within range) and TNF-α (100% of all measured values within range).

#### Interleukin-10

**Fig. 3:**
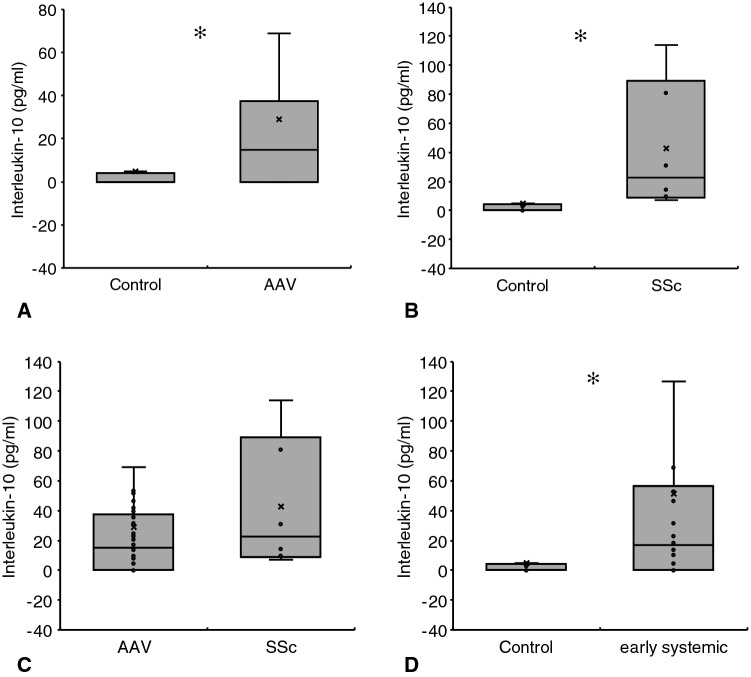
Serum IL-10 in controls versus AAV and SSc. The cytokine was higher in both, AAV (**a**) and SSc (**b**), as compared to controls, but did not differ between AAV and SSc (**c**). Subjects with early systemic AAV showed higher serum IL-10 than controls (**d**) (Kolmogorov–Smirnov test for normality: IL-10 Control—*p* = 0.004; IL-10 AAV—*p* < 0.001; IL-10 SSc—0.18; IL-10 early systemic − < 0.001; Data as media ± Q1/Q3; ✻*p* < 0.05—for exact *p* values see text)

Our analysis showed higher Interleukin-10 serum levels in AAV patients as compared to healthy subjects (29 ± 14.7 vs. 4.6 ± 4.4 pg/ml; *p* = 0.004). The cytokine did, however, not differ between AAV and SSc (29 ± 14.7 vs. 42.7 ± 46.5 pg/ml; *p* = 0.225) but SSc subjects showed higher IL-10 than healthy controls (42.7 ± 46.5 vs. 4.6 ± 4.4 pg/ml; *p* < 0.001). The cytokine did not differ between AAV individuals with different disease stages, according to the 2007 published EULAR recommendations [13]. However, AAV patients with early systemic vasculitis displayed higher serum IL-10 than healthy controls (51.2 ± 40.7 vs. 4.6 ± 4.4 pg/ml; *p* = 0.005). Also, serum IL-10 did neither differ between AAV patients with and those without relapsing vasculitis or between individuals with and those without renal or upper/lower respiratory involvement, respectively. Regarding the immunosuppressive therapy, IL-10 levels were comparable in RTX treated and RTX untreated patients but lower in subjects undergoing treatment with RTX and cyclophosphamide combined. Figure 3 summarizes the essential results of all IL-10 analyses.

#### Interleukin-33

In comparison to healthy controls, AAV-patients showed significantly elevated serum IL-33 concentrations (165.8 ± 64.4 vs. 71.7 ± 79.8 pg/ml; *p* < 0.001). Similar observations were made in SSc (365.7 ± 187.3 vs. healthy controls 71.7 ± 79.8 pg/ml; *p* = 0.001). IL-33 did also significantly differ between AAV and SSc (*p* = 0.009). PR3+ individuals did not differ from controls, and the difference between PR3+ and MPO+ subjects was only close to the level of significance (*p* = 0.06).

**Fig. 4:**
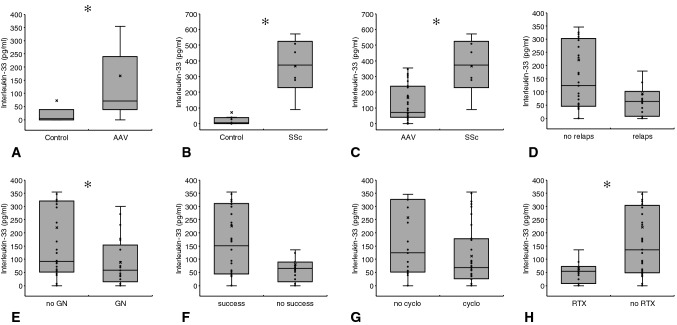
Serum IL-33 analyses. As compared to controls, the cytokine was higher in both, AAV and SSc (**a** and **b**) and differed between the two diseases (**c**), as well. Lower levels were detected in necrotizing GN (**e**) and RTX treated subjects (**h**). All other differences were not statistically significant (**d**, **f**, **g**) (Kolmogorov-Smirnov test for normality: IL-33 Control—*p* < 0.001; IL-33 AAV—*p* < 0.001; IL-33 SSc—*p* = 0.821; IL-33 no relaps—*p* < 0.001; IL-33 relaps—*p* < 0.001; IL-33 no GN—*p* < 0.001; IL-33 GN—*p* < 0.001; IL-33 no success—*p* < 0.001; IL-33 success—*p* < 0.001; IL-33 no cyclo(phosphamide)—*p* < 0.001; IL-33 cyclo(phosphamide)—*p* < 0.001; IL-33 no RTX—*p* < 0.001; IL-33 RTX—*p* < 0.001; Data as media ± Q1/Q3; ✻*p* < 0.05—for exact *p* values see text)

Regarding the AAV cohort alone, IL-33 did not differ between relapsing and non-relapsing disease (92.7 ± 48.1 vs. 221.2 ± 105.9 pg/ml; *p* = 0.1), but it was lower in subjects with renal involvement as compared to those without such a manifestation (87.9 ± 41.8 vs. 220.3 ± 103.7 pg/ml; *p* = 0.04). Patients with versus without upper/lower respiratory involvement did not differ in serum IL-33 concentrations. If related to the immunosuppressive treatment regimens, one additional difference appeared: patients undergoing RTX treatment displayed lower IL-33 levels than subjects without such therapy (64.4 ± 39.8 vs. 221.1 ± 93.6 pg/ml; *p* = 0.01). Figure 4 summarizes the essential results of all IL-33 analyses.

#### Tumor necrosis factor-alpha (TNF-α)

Serum TNF-α concentrations did not differ between AAV and controls (7.7 ± 5.7 vs. 3.3 ± 1.9 pg/ml; *p* = 0.34) but they were significantly lower in AAV than in SSc (7.7 ± 5.7 vs. 28.6 ± 35.9 pg/ml; *p* < 0.0001). The latter observation was made in both types of AAV, PR3 + and MPO + vasculitis (7.2 ± 5.6 vs. 28.6 ± 35.9 pg/ml, *p* < 0.0001 and 9.6 ± 6.1 vs. 28.6 ± 35.9 pg/ml; *p* < 0.004). In SSc, serum TNF-α was higher than in controls (28.6 ± 35.9 vs. 3.3 ± 1.9 pg/ml; *p* < 0.0001). Out of all analyzed parameters, TNF-α showed the fewest associations with any parameters, including disease stages and renal or respiratory involvement. Nevertheless, successful induction therapy was associated with higher TNF-α levels (9.2 ± 6.5 vs. 5.6 ± 3.5 pg/ml; *p* = 0.02).

#### Soluble CD40 Ligand (sCD40L)

**Fig. 5:**
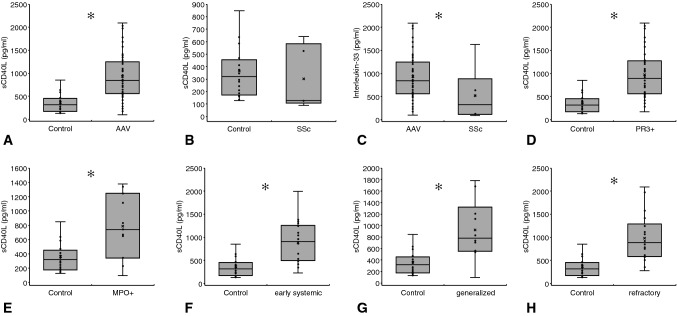
Serum sCD40L analyses. Soluble CD40L was higher in AAV as compared to controls (**a**) and to SSc (**c**). It did not differ between SSc and controls (**b**). PR3+ and MPO+ individuals showed elevated sCD40L in comparison to controls (**d** and **e**). If compared to the controls, AAV subjects with either early systemic (**f**) or generalized (**g**) or refractory (**h**) disease displayed higher serum sCD40L (Kolmogorov-Smirnov test for normality: sCD40L Control—*p* = 0.004; sCD40L AAV—*p* = 0.032; sCD40L SSc—*p* = 0.134; sCD40L PR3—*p* = 0.057; sCD40L MPO—*p* = 0.915; sCD40L early systemic—*p* = 0.089; sCD40L generalized—*p* = 0.949; sCD40L refractory—*p* = 0.167; Data as media ± Q1/Q3; ✻*p* < 0.05—for exact *p* values see text)

sCD40L was higher in all AAV patients than in controls (935.2 ± 141.7 vs. 364.3 ± 106.7 pg/ml; *p* < 0.001). PR3 + and MPO + individuals displayed higher serum sCD40L levels than controls (976.4 ± 165.1 and 785.3 ± 299.7 vs. 364.3 ± 106.7 pg/ml; *p* < 0.001 and *p* = 0.014) (Fig. 5). Serum concentrations differed between SSc and AAV (522.4 ± 618.2 vs. 935.2 ± 141.7 pg/ml; p = 0.04) but not between SSc and controls (*p* = 0.74) (Fig. 5). As observed in all other cytokines, sCD40L did not significantly vary in the specific disease stages, according to the 2007 published EULAR recommendations [13]. However, we identified differences between healthy controls and certain disease stages: controls vs. early systemic—364.3 ± 106.7 vs. 889.6 ± 227.4 pg/ml; *p* < 0.001; controls vs. generalized—364.3 ± 106.7 vs. 924.7 ± 374.9 pg/ml; *p* = 0.003; controls vs. refractory—364.3 ± 106.7 vs. 978.4 ± 257.1 pg/ml; *p* < 0.001. Further positive or negative associations were not identified for sCD40L. Figure 5 summarizes the essential results of all sCD40L analyses.

We would finally like to mention that the BVAS did not correlate with any of the four cytokines analyzed, neither in MPO+ nor in PR3+ AAV subjects.

## Discussion

The current study aimed to investigate serological abnormalities in AAV subjects. The principal goal was to identify new candidates for serological testing, thus to widen the currently limited spectrum of diagnostic and prognostic serum markers in these serious autoimmune-mediated conditions. The most intriguing result of our study were all the findings which must be considered as negative or absent: numerous serum candidate cytokines were not detectable at all/fell under the lower detection limit of the assay (IL-1β, IL-4, IL-6, IL-17A, IL-17F, IL-21, IL-22, IL-23, IL-25, IL-31, INF-γ). Only four parameters finally fulfilled the criterium that at least 50% of all individual measurements were above the lower detection limit of the assay: IL-10, IL-33, TNF-α, and sCD40L. Although we analyzed clinical aspects of GPA subjects as well, the discussion section will exclusively focus on serological characteristics.

IL-10 substantially promotes the immunoglobulin switch in B cells. It has been identified as an essential element in B cell-mediated autoimmunity [14]. Lepse and colleagues [15] found reduced numbers of B regulatory cells in AAV, while IL-10 levels did not differ between AAV subjects and healthy controls. All AAV subjects and particularly those with early systemic disease displayed higher IL-10 than controls; also, we found higher IL-10 in relapse-free subjects without reaching the level of significance. Comparable observations were made by Ohlsson et al., who detected significantly lower IL-10 in individuals suffering from any relapse within the first 3 months after successful remission induction [16]. Hruskova and colleagues found lower in-remission IL-10 to be associated with a higher relapse probability [17]. Finally, higher IL-10 has been identified to go in parallel with an increased risk for future relapses [18]. Thus, we support the hypothesis of Ohlsson et al. [16] who proposed IL-10 as a suppressor of latent disease activity as it may persist even during complete remission.

As pointed out earlier, Interleukin-33 belongs to the IL-1 cytokine family. It is produced by stromal, epithelial, and endothelial cells, respectively [19]. Its effects include either propagation or inhibition of inflammatory processes, depending on the respective microenvironmental circumstances [20]. Our study revealed higher IL-33 in all AAV subjects. Renal involvement was associated with lower IL-33; the cytokine did in contrast not differ between apparent and absent upper/lower respiratory involvement. Renal manifestations belong to the most severe complications in AAV. One may argue that during renal injury and repair, apoptosis rather than necrosis is the predominant mechanism of cell damage. Apoptosis has been proposed to reduce IL-33 availability by caspase-mediated IL-33 degradation [21]. Our study revealed lower IL-33 levels to be associated with more frequent use of RTX.

The pro-inflammatory cytokine Tumor Necrosis Factor-alpha (TNF-α) mediates diverse processes involved in the inflammatory response. They include MHC induction, macrophage activation, leukocyte-endothelial adhesion, and increased hepatic synthesis of acute-phase proteins [22]. Our study revealed only a few differences in serum TNF-α between the respective subgroups. TNF-α was higher in SSc as compared to AAV and controls. The second finding regarding this particular cytokine was increased TNF-α levels in subjects undergoing successful induction therapy. We suppose that the relatively low concentrations in PR3+ and MPO+ AAV ensue from pre-established steroid treatment which has been performed in all individuals. Whether TNF-α indeed plays a pathogenic relevant role in AAV can be doubted, although earlier open-label studies showed beneficial effects of blocking the substance in vivo [23]. Today, anti-TNF-alpha agents are not even recommended in refractory disease courses [24].

CD40 Ligand belongs to the TNF family; it is expressed on activated CD4+ T cells, B cells, and platelets. In inflammatory states, de novo expression of the protein occurs on monocytes, natural killer cells, mast cells, and basophils [25]. Soluble CD40L (sCD40L) on the other hand, has been proposed as a marker of B cell activation [26]. Our analysis showed significantly higher sCD40L in AAV, and thereby in both PR3+ and MPO+ individuals. Remarkably, AAV subjects displayed elevated sCD40L in all disease stages without any differences between individual stages, respectively. Thus, high levels of the cytokine most likely reflect activation of the cellular immune response in general rather than disease- or stage-specific phenomenons. We failed to show any correlation between ANCA titer and sCD40L as it has been demonstrated by Tomasson and colleagues [27]. We also did neither detect differences between patients with versus without successful (re-) induction therapy nor between those with versus without renal involvement. Further significant associations for sCD40L were missing as well. Therefore, we currently do not believe in any substantial diagnostic/prognostic value of sCD40L in AAV.

## Conclusions

Serum IL-10 may potentially serve as a marker of early systemic AAV. Serum IL-33 may help to identify subjects with a higher risk for necrotizing GN. Further studies must focus on longitudinal dynamics of these cytokines in AAV of different severity/activity.

## Limitations

The most relevant flaw of the study is the current lack of longitudinal data. Particularly the aspect of cytokine (Il-10 and -33) dynamics over time, from the moment of the initial diagnosis and before the initiation of any treatment until incomplete or complete remission needs to evaluated systematically. One may also argue that the exclusive inclusion of cytokines, of which at least 50% of all measured values were above the lower detection limit of the assay is a limitation. Without this rule, other significant differences occur as well (e.g., IL-6 and IFN-g).
